# Robust Spectral Clustering Using Statistical Sub-Graph Affinity Model

**DOI:** 10.1371/journal.pone.0082722

**Published:** 2013-12-26

**Authors:** Justin A. Eichel, Alexander Wong, Paul Fieguth, David A. Clausi

**Affiliations:** Department of Systems Design Engineering, U. of Waterloo, Waterloo, Canada; Universidad de Zarazoga, Spain

## Abstract

Spectral clustering methods have been shown to be effective for image segmentation. Unfortunately, the presence of image noise as well as textural characteristics can have a significant negative effect on the segmentation performance. To accommodate for image noise and textural characteristics, this study introduces the concept of sub-graph affinity, where each node in the primary graph is modeled as a sub-graph characterizing the neighborhood surrounding the node. The statistical sub-graph affinity matrix is then constructed based on the statistical relationships between sub-graphs of connected nodes in the primary graph, thus counteracting the uncertainty associated with the image noise and textural characteristics by utilizing more information than traditional spectral clustering methods. Experiments using both synthetic and natural images under various levels of noise contamination demonstrate that the proposed approach can achieve improved segmentation performance when compared to existing spectral clustering methods.

## Introduction

Spectral clustering is a powerful tool for machine learning designed to cluster similar data, making image segmentation a popular application. Zhang et al. [1] segment plant types from SAR images using a ensemble-learning and spectral clustering. Lui and Jia [2] apply texture characteristics and Markov random fields with spectral clustering ensembles to segment land types from SAR data. Sundaram and Keutzer [3] applied spectral clustering with GPU parallelization to segment people and objects from video sequences. As a medical application, Ge et al. [4] apply spectral clustering to segment the corpus callosum white matter fibers connecting the left and right brain hemispheres in diffusion tensor imaging (DTI). Carcassoni and Hancock have developed techniques that improve spectral clustering applied to correspondence matching improving the robustness of matching similar structures, demonstrated on object clustering within images [5], [6].

For each data point, or each pixel in the domain of image segmentation, a similarity matrix or, more generally, an affinity matrix, 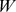 is constructed. Each element in the matrix, 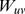, represents how similar pixel 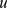 is to pixel 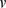. The similarity matrix is transformed into an eigenvector domain, allowing the derived eigenvectors to provide a means to identify the most significant features within an image. Ideally, each salient image region transforms into a cluster of data points in the eigenvector domain. Using a clustering algorithm such as k-means or hierarchically clustering, the resulting clusters are identified and labeled. As long as there are a sufficient number of good measurements, spectral clustering can be very effective at segmenting an image.

Two important challenges faced when applying spectral clustering to achieve image segmentation is dealing with the presence of i) bad measurements and/or noise-corrupted measurements, and ii) textural characteristics and variations within image regions. Hong et al. [7] encounter this situation in underwater sonar images. In such situations, the pixels within the image may be misclassified using existing spectral clustering approaches given their heavy reliance on the affinity between connected nodes in constructing the affinity weighted graph; the pixel intensities can differ significantly due to noise contamination and textural characteristics and variations. While Hong et al. improve spectral clustering through morphological wavelet transformations for sonar images, they require additional post-processing to the spectral clustering framework and do not account for any textural characteristics within the image.

Expanding on pixel-to-pixel nodal affinity, Pardo [8] touches on the idea of using region statistics to better classify the edges of regions in noisy images. For each region, Pardo calculates the standard deviation and mean, allowing regions with similar statistics to be combined into a single region. Comparing two region statistics assumes that each region has uniform intensity, which does not account for textures or region structures that can be detected through a sub-graph method. Therefore, a means to provide more robust image segmentation under heavy noise contamination and in situations where regions exhibit textural patterns and variations is desired.

The main contribution of this study is the introduction of a robust spectral clustering approach to segment images under the presence of heavy noise contamination as well as the presence of textural patterns and variations in image regions. Instead of relying on the node affinity between connected nodes in the construction of the affinity weighted graph, the proposed approach utilizes the concept of statistical sub-graph affinity between connected nodes. By taking into consideration the spatial-intensity relationships within sub-graphs, the assumption is that the statistical affinity between sub-graphs is less sensitive to noise, which is random in nature and does not typically exhibit such structured relationships, and better characterizes the underlying textural characteristics.

By themselves sub-graphs are not unique; Luo et al. [9] also utilize sub-graphs, they improve robustness by clustering graphs relating to the structure of objects, applied during the clustering step of spectral clustering. Instead our method applies sub-graphs to extend the affinity metric to include a comparison of the neighbouring regions; the sub-graph affinity utilizes region statistics before the clustering step so that similar regions, instead of similar nodes, can be clustered more robustly Our work focuses on extending the nodal affinity step of spectral clustering to a statistical sub-graph affinity.

The rest of this paper is organized as follows. In Methods, we explain the methodology of the proposed statistical sub-graph affinity modeling approach. In Results, experimental results using both synthetic and natural images under various levels of noise contamination are presented and discussed. Finally, conclusions are drawn and potential future work are discussed in the remaining two sections.

## Methods

In this section, the methodology behind the proposed statistical sub-graph affinity model for spectral clustering is discussed in detail. First, the traditional node affinity model for spectral clustering is described for context. Second, the proposed statistical sub-graph affinity model, which is designed to better account for noise contamination and textural characteristics and variations within the image being segmented, is presented.

### Node Affinity Model for Spectral Clustering

In the node affinity model for spectral clustering, similarity metrics measure the difference between two nodes and are typically scaled between zero (dissimilar) and one (similar). Given an image 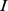 of size 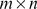, a given pixel 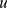 has a node measurement 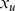 that directly corresponds to a grey-scale intensity,
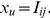
(1)Each edge, 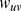, in the affinity graph represents the node affinity, or similarity between two node measurements, 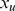 and 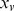. The affinity graph can also be represented as a weighted adjacency matrix 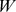, where the elements are equal to 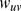. In [10], [11], the weight between two connected nodes 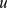 and 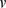 is computed based on their node affinity,
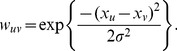
(2)


Using the affinity graph with weights defined in Eq. (2), many authors have contributed variations that produce different clustering results depending upon circumstances [12]–[22]. Perona and Freeman [12] calculated the eigenvectors of 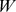 and applied thresholding to the principal eigenvector in order to separate two classes of data.

Instead of working with 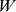 directly, Shi and Malik [13] computed the unnormalized Laplacian,
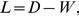
(3)where 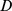 is a diagonal matrix defined as
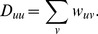
(4)Shi and Malik applied thresholding to classify data points into classes based on multiple principal eigenvectors. The matrix 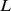 associates the number of connected components in the graph, 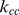, with the multiplicity of the 0 eigenvalue of 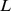. This allows the number of connected components for a particular system to be found. This approach was subsequently extended to incorporate contour and texture information [14].

Scott and Longuet-Higgins [15] extended the thresholding methods with more generalized clustering techniques to improve clustering results. Ng et al. [16] introduced normalized Laplacian matrices,
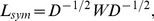
(5)and clustered the principal eigenvectors of 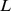 using the k-means clustering algorithm. Variations of this method also include defining an asymmetric normalized Laplacian as
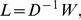
(6)which has properties similar to that of a random walk. An in-depth discussion of the various connected-graphs and affinity matrices is provided by Chung and Mohar [17]–[19].

One of the key limitations with the node affinity model for spectral clustering is that, by only taking into account the intermediate similarity between two node measurements, the information available for assessing affinity is relatively limited and as such can be sensitive to the presence of noise. Furthermore, by only taking the individual node measurements into account, the node affinity model provides a relatively poor characterization of the textural characteristics within the image, thus limiting the ability to account for situations where the image is characterized by highly textured segmentations as well as textural variations. Therefore, the main motivation behind the proposed statistical sub-graph affinity model is to counteract the uncertainty associated with the image noise and textural characteristics by utilizing more information than the node affinity model.

### Statistical Sub-graph Affinity Model

The proposed model attempts to improve the robustness of spectral clustering for situations characterized by heavy noise contamination by replacing the use of node affinity described in Eq. (2) with the concept of statistical sub-graph affinity.

In order to create the affinity matrix, the connectivity of each node to every other node must be represented in a connectivity graph, 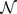. When dealing with large images, memory and computer resources need to be conserved; to do so, 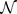 is implicitly constructed from the graph kernel 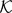. The kernel weights, elements of 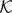, represent the degree of connectivity between a node and its neighbor.

**Figure 1:**
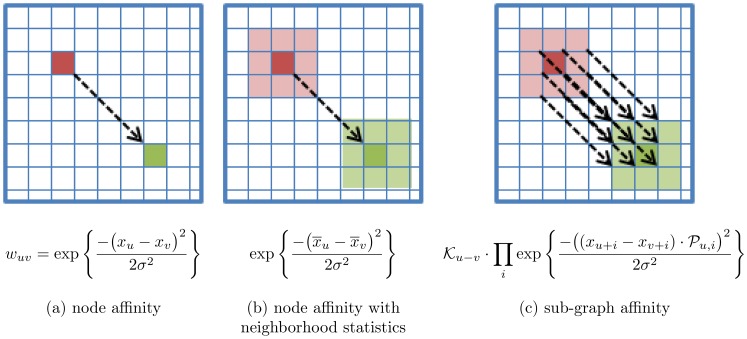
Comparison of node affinity to sub-graph affinity. This figure illustrates a pixel-to-pixel rectangular sub-graph specific to implementation for an image and the corresponding edge weight equations. (a) For node affinity, each node, 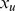, (red) is compared to a neighbouring node, 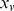 (green), for all nodes in the local neighborhood, 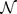. (b) If neighborhood statistics, such as a mean or weighted average, were applied as preprocessing to the data or image, the resulting similarity metric would contain regional statistics and would be more robust to noise with the cost of losing fine details such as edges and textures. (c) Rather than calculating the similarity of two regions from a single statistic, as in (b), sub-graph affinity calculates the similarity of two regions by calculating the similarity of each corresponding element within the regions, improving robustness to noise while preserving fine details of the region. Using sub-graph affinity, two regions with the same average intensity, but with differing textures, can be classified as two different classes.

The novelty of the proposed method is the introduction of a statistical sub-graph affinity model to replace the node affinity model; instead of comparing 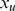 to 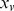 for all 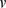 in the neighborhood of 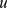, the sub-graph 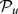 is compared against each sub-graph 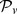, which is illustrated in Fig. 1.

A statistical mathematical description of the process is required to understand how sub-graphs can improve classification accuracy. Given a node 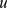 belonging to class 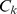, the nodal features, 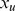, can be modeled as the random process, 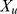, affected by independent and identically distributed (i.i.d.) noise, 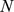:
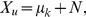
(7)where 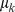 is the mean of all nodal features belonging to class 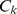.

The effect of noise is reduced by incorporating neighborhood information contained within a sub-graph, 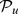, which stores the connectivity of node 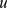 to its neighbors. When using node affinity, the probability that that two nodes, 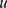 and 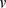, belong to the same class, 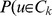 and 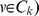, denoted as 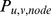, is the probability that the difference between 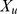 and 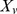 is equal to zero,
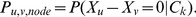
(8)Since 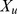 and 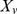 are i.i.d, then 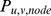 can be determined using the Laplace distribution,
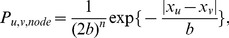
(9)where 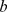 is the scaling factor associated with 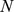. Compared to node affinity, sub-graph affinity suppresses noise. The resulting sub-graph affinity probability, 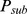, is equal to,
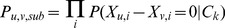
(10)

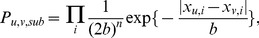
(11)where 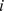 is the index of each element in 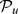 and corresponding element in 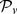. The more similar values in each sub-graph, the smaller 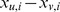 becomes, increasing the probability of 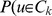 and 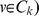; the local neighborhood information suppresses the noise.

Furthermore, the use of sub-graphs allows the spatial-intensity relationships between nodes within a sub-graph to be preserved. This intra-sub-graph spatial-intensity relationship preservation is important as it allows for improved modeling and representation of textural characteristics within the image. As such, the proposed statistical sub-graph affinity model accounts for uncertainties due not only to noise contamination but also to textural characteristics and variations within the image.

Given the proposed statistical sub-graph affinity model, one can now incorporate this model into the spectral clustering framework by extending upon the weighting metric introduced in Eq. (2). The density of 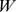 depends primarily on the complexity of 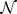 and is independent of 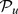. The values of 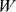, which depend on the kernel weights and the statistical sub-graph affinity, can be defined as
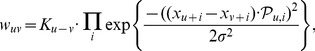
(12)for nodes 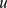 and 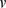. The weights associated with 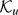 and 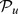 are also introduced to allow spatial weighting if so desired.

**Figure 2:**
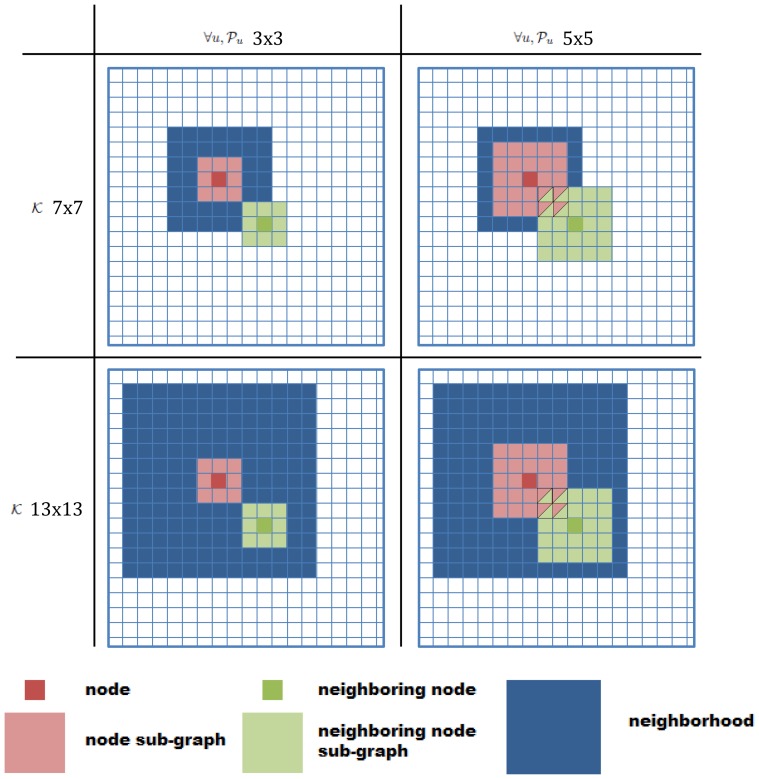
Effects of graph kernel size and sub-graph kernel size on neighbourhoods and algorithm complexity. The effects of increasing the size of the graph kernel, 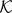, and increasing the size of the sub-graph kernel, 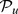, are illustrated using a two-dimensional example above. As the size of 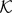 increases, from top to bottom, the size of the neighbourhood (solid dark blue) surrounding 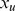 (dark red) increases. As the size of 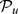 increases, from left to right, the size of the sub-graphs (light red and light green) increases. Increasing the size of 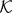 decreases the sparsity of *W* and increasing the size of 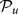 increases the computational complexity of calculating 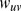, but only moderately.

Fig. 2 helps illustrate how 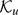 and 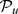 affect the size of the neighbourhood and the computational complexity of Eq. (12). Increasing the size of 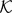 increases the size of the neighborhood, and consequently, the number of 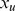 and 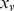 similarly calculations, which results in decreasing the sparsity of 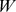. Increasing the size of 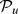 has no effect on the neighborhood size and, consequently, does not effect the density of 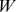. Rather increasing the size of 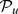 increases the computational complexity of calculating 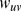, but only moderately, as the difference of 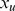 and 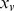, which can be cached after initial calculation. The size of 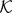 has a greater impact on the overall computational complexity than the size of 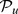.

Kernel selection allows the algorithm to become rotationally invariant, by selecting radially symmetric kernels. The kernels can also encode distance information so that the sub-graph weights or connectivity can be a function of spatial pixel distances, i.e., the intensity difference of corresponding pixels can be weighted based on spatial distance from the center of the sub-graph. The sub-graph affinity model described in Eq. (2) compares the statistical spatial-intensity relationships between the sub-graph around 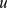 with the sub-graph of 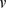. By incorporating the spatial-intensity relationships within sub-graphs into the construction of the affinity weighted graph, the constructed graph is less sensitive to the influence of random noise on the variations of individual pixel intensities, as well as better accounts for textural characteristics. As a consequence, the use of statistical sub-graph affinity should be more robust to noise as well as textural variations.

**Figure 3:**
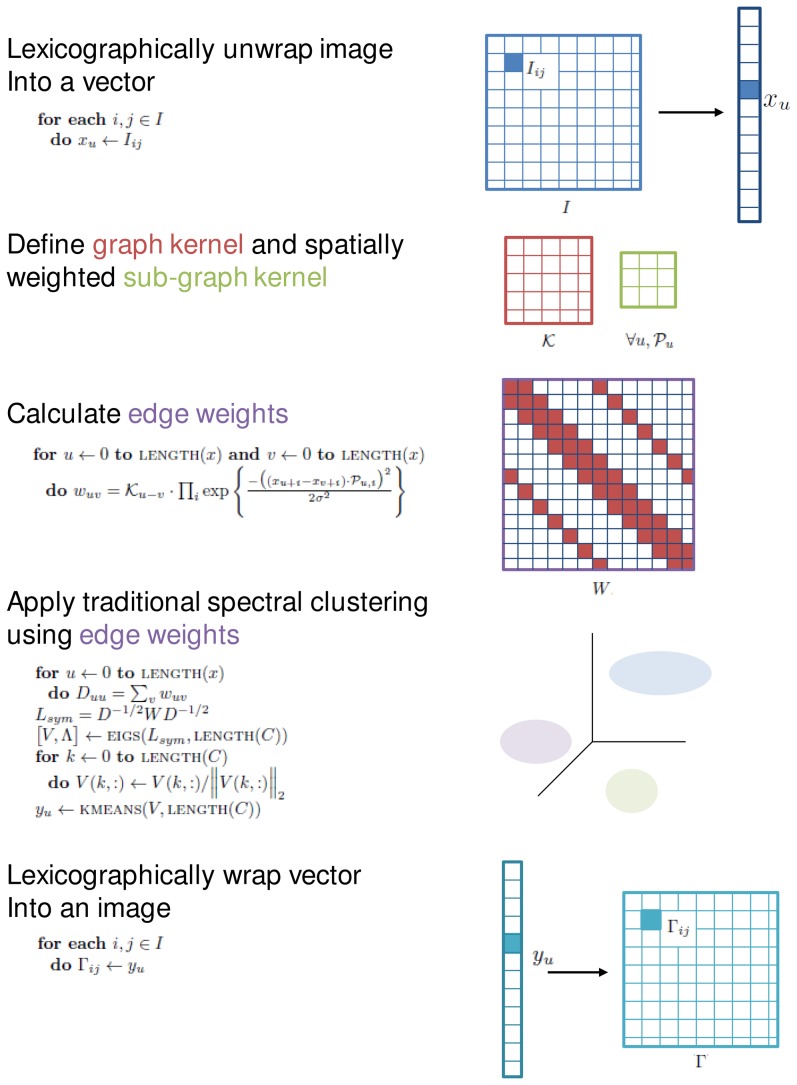
Overview of spectral clustering algorithm with statistical sub-graph affinity. The image is lexicographically unwrap into a vector, spatially weighted kernels 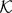 and 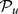 are constructed, edge weights are calculated, and traditional spectral clustering techniques are applied before the labelled data is wrapped back into a labelled image. Although the kernels are illustrated as matrices above, the kernels can be constructed as an arbitrary graph.

Using Eq. (1) through (5), and the proposed edge weights, Eq. 12, the entire algorithm is presented in Fig. 3. The first step is to lexicographically unwrap the each pixel (or region), 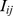, in 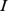 into a vector 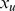. The second step is to define the kernels 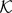 and 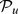. The third step is to construct the edge weights, 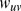, from a pixel-to-pixel (or region-to-region) comparison, 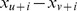, for all pixels (or regions) in 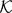. The kernel 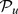 applies spacial weighting to pixels (or regions) surrounding 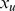. The fourth step is to apply any existing spectral clustering technique that can use our edge weights, 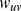. The result of the spectral clustering technique is the labelled pixels (or regions), 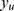. The final step is to wrap the vector 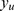 into image pixels (or regions), 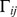.

## Results

To investigate the performance of the proposed approach for segmenting images under heavy noise contamination, the proposed statistical sub-graph affinity model was incorporated into the Ng-Jordan-Weiss spectral clustering method [16]. For comparison purposes, the original Ng-Jordan-Weiss [16], Shi Malik [13], and Scott Longuet-Higgins [15] methods the node affinity model was also tested. Spectral clustering was executed using MATLAB and custom built MEX libraries for efficiently constructing symmetric normalized Laplacian adjacency matrix for each trial.

**Figure 4:**
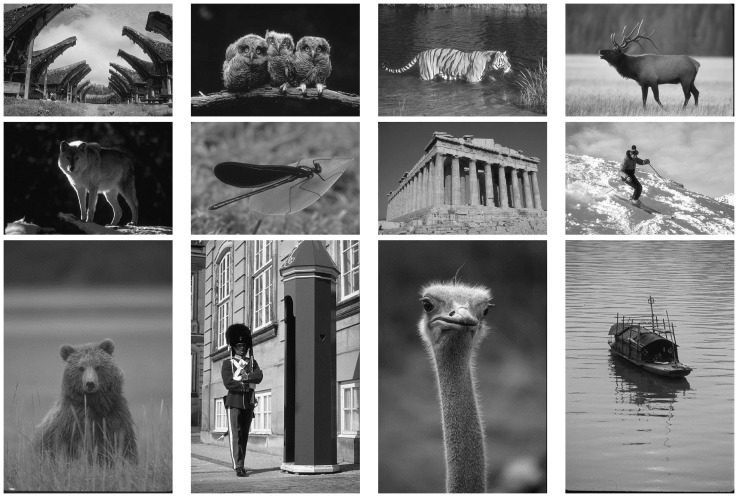
Real-world images used for testing. The performances of spectral clustering using node affinity and sub-graph affinity were evaluated using the above test images, reproduced from the Berkeley Segmentation Dataset [23].

Even though our framework can segment images larger than 1024×1024 pixels, test image sizes ranged from 51×51 to 250×250 pixels so that results can be compared to existing methods. The test image set included several synthetic images, for initial testing, and 20 images from the Berkeley Segmentation Dataset [23], Fig. 4, for thorough analysis. The trials evaluated the graph kernel size iterating them over the following sizes, 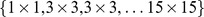; the sub-graphs were iterated through the same set of sizes. Both uniform and Gaussian weighted radial graph and sub-graph kernels were tested. The trials tested each combination of graph and sub-graph kernel sizes.

The PSNR ranged from infinity to 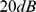 as images exposed to Gaussian, speckle, and salt and pepper noise were segmented. For illustrative purposes, rectangular lattices are employed for the sub-graph affinity model in the implementation, although other graph structures may also be used. Best results were obtained when 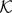 is uniform and has the largest size possible and 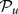 is Gaussian with a relatively small size in comparison to 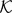.

### Quantitative Assessment

To quantitatively evaluate the performance of the proposed statistical sub-graph affinity model in the presence of noise, the f1-measure was computed on the segmentation results produced by both the node affinity model and the statistical sub-graph affinity model:
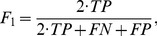
(13)where 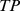, 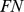, and 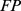 denotes the number of true positives, false negatives, and false positives, respectively, in the segmentation results.

**Figure 5:**
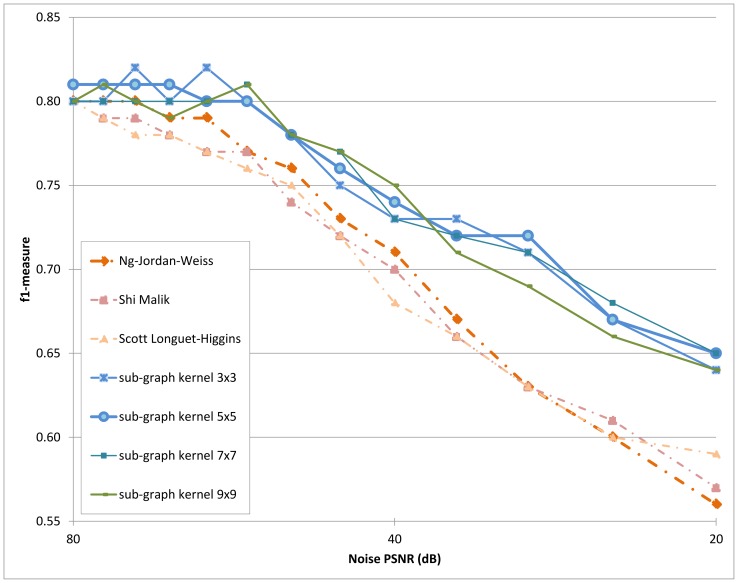
Segmentation results show how well the sub-graph affinity model outperforms several node affinity models. For all tests, the proposed statistical sub-graph affinity model performs as well as or outperforms the node affinity model. The dotted lines represent the mean f1-measure for the corresponding nodal affinity model. The solid lines represent the mean f1-measure for the statistical sub-graph affinity models associated with sub-graph kernel sizes of 3×3 through 9×9. The proposed sub-graph affinity model outperforms existing nodal affinity models at low PSNR.

Fig. 5 and Table 1 present the f1-measure results of spectral clustering with the node affinity model and with the statistical sub-graph affinity model compared to ground truth provided by the Berkeley Segmentation Dataset [23] against existing spectral clustering methods. Spectral clustering using the proposed sub-graph affinity model achieve similar f1-measures to spectral clustering results for existing nodal affinity model. However, in the presence of noise, even a 3×3 statistical sub-graph affinity model shows immediate improvements over existing methods. Since the statistical sub-graph affinity model uses the spatial-intensity structural characteristics within sub-graphs, the statistical sub-graph affinity model is less sensitive to random noise that does not have such characteristics, and better characterizes the underlying textural characteristics in an image, thus leading to improved image segmentation accuracy.

**Table 1:** The effect of noise on spectral clustering f1-measures is illustrated.

	Ng-		Scott	proposed						
PSNR	Jordan-	Shi	Longuet-	kernel size						
(db)	Weiss	Malik	Higgins	3	5	7	9	11	13	15
80	0.80	0.80	0.80	0.80	**0.81**	0.80	0.80	0.80	0.79	0.80
75	0.80	0.79	0.79	0.80	**0.81**	0.80	**0.81**	0.80	**0.81**	**0.81**
70	0.80	0.79	0.78	**0.82**	0.81	0.80	0.80	**0.82**	0.80	0.80
65	0.79	0.78	0.78	0.80	**0.81**	0.80	0.79	**0.81**	0.80	0.80
60	0.79	0.77	0.77	**0.82**	0.80	0.80	0.80	0.81	0.81	0.81
55	0.77	0.77	0.76	0.80	0.80	**0.81**	**0.81**	0.80	0.79	**0.81**
50	0.76	0.74	0.75	0.78	0.78	0.78	0.78	**0.80**	**0.80**	0.79
45	0.73	0.72	0.72	0.75	0.76	0.77	0.77	**0.78**	0.77	0.77
40	0.71	0.70	0.68	0.73	0.74	0.73	**0.75**	**0.75**	0.73	**0.75**
35	0.67	0.66	0.66	**0.73**	0.72	0.72	0.71	0.70	0.72	0.72
30	0.63	0.63	0.63	0.71	**0.72**	0.71	0.69	**0.72**	0.67	0.70
25	0.60	0.61	0.60	0.67	0.67	0.68	0.66	**0.69**	0.67	0.67
20	0.56	0.57	0.59	0.64	**0.65**	**0.65**	0.64	0.64	0.64	**0.65**

The proposed sub-graph affinity model performs as well as existing spectral clustering methods when PSNR is high. When the PSNR is low, the sub-graph affinity model outperforms f1-measures for the Ng-Jordan-Weiss, Shi Malik, and Scott Longuet-Higgins nodal affinity models.

### Visual Assessment

**Figure 6:**
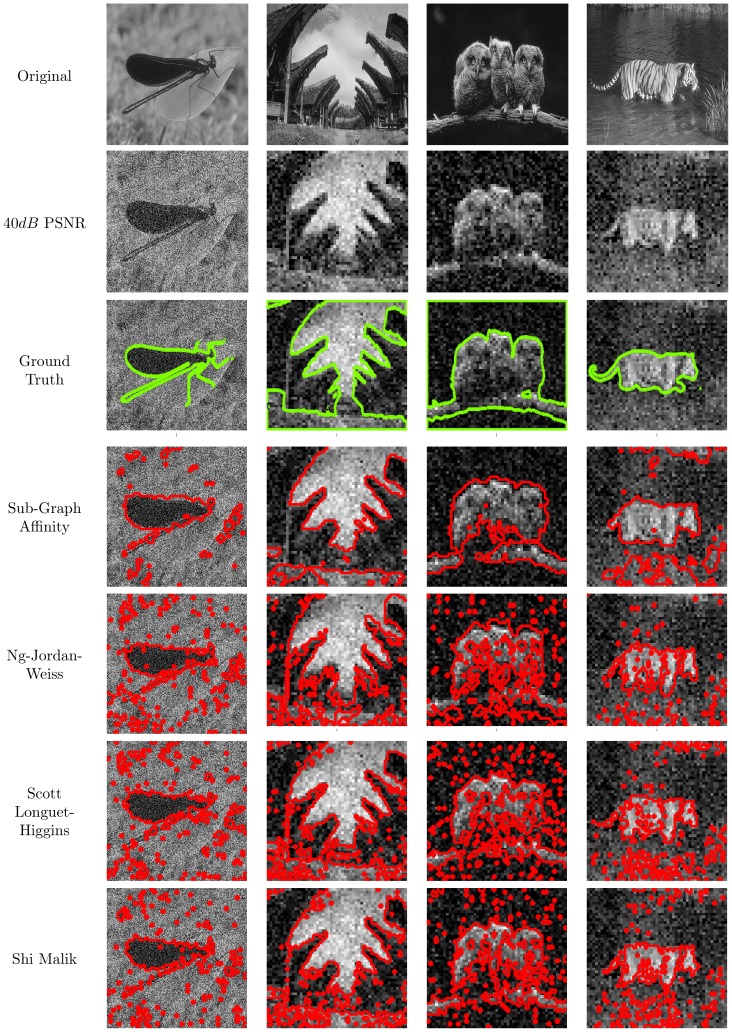
Object boundaries resulting from sub-graph a_nity and node a_nity segmentation compared to ground truth. The object boundaries resulting from the segmentation process are illustrated in red; the ground truth boundaries are illustrated in green. In the presence of noise, spectral clustering using the proposed statistical sub-graph a_nity model produces more robust results than the other tested methods which use the node a_nity model. Noise tolerance is most evident in the ‘owls’ image, where the segmentation results produced by the proposed statistical sub-graph a_nity model consists of large homogeneous segments, whereas that produced by the other tested methods using a node a_nity model contain many small noisy segments. Furthermore, the proposed statistical sub-graph a_nity model better handles the presence of textural characteristics within the images, as evident in the ‘tiger’ image; the body of the tiger is identi_ed as a single segment by the proposed model.

Fig. 6 visually shows segmentation results generated with the use of the node affinity model and the proposed statistical sub-graph affinity model in the presence of noise. It can be observed that the proposed model is noticeably more robust to the presence of noise. This is most evident in the ‘circle-triangle’ image, where the segmentation results produced by the proposed model consists of large homogeneous segments compared to the node affinity model, which consists of many small noisy segments. Furthermore, it can be observed that the proposed model better handles the presence of textural characteristics within the images. This is most evident in the ‘tiger’ image, where the proposed model allows the body of the tiger to be identified as a single segment, whereas the node affinity model identifies the body as multiple smaller segments.

### Conclusion

In this study, a robust spectral clustering strategy is introduced to better handle situations characterized by heavy noise, as well as situations containing textural characteristics and variations. The concept of statistical sub-graph affinity was introduced for constructing the affinity weighted graph based on the spatial-intensity relationships between connected nodes. Experimental results obtained from both synthetic and natural images under varying noise levels show that the proposed approach produces more robust segmentation results when compared with existing spectral clustering methods.

As a result of the positive experiments in this study, we propose that further investigation of the sub-graph affinity model be explored for the purpose of image segmentation. Notably, we propose investigation of more data-adaptive sub-graph models that change based on the underlying structural characteristics. In addition to the general independent and identically distributed noise model presented in this paper, other application dependent statistical models can be developed in a similar manner since spectral clustering for each application can be improved with better statistical models. Further, while this study illustrates sub-graph affinity in image segmentation, future studies can extensively apply the proposed method to video segmentation, or more generally any temporal segmentation, and to highly dimensional data structures.

## Discussion

The robustness of the proposed statistical sub-graph affinity model when dealing with the presence of noise stems mainly from two key factors. The first key factor is the fact that the proposed statistical sub-graph affinity model directly incorporates a statistical noise model when characterizing the sub-graphs as random processes, thus better handling the uncertainty associated with the presence of noise. The second factor is the fact that statistical sub-graph affinity model incorporates neighborhood connectivity when characterizing the sub-graphs, thus taking advantage of neighborhood information to compensate for uncertainties due to noise. The robustness of the proposed statistical sub-graph affinity model when dealing with images characterized by heterogeneous texture characteristics stems from the fact that textural patterns, which is defined by local neighborhood connectivity and interactions, are better modeled using a sub-graph affinity model (which incorporates such local connectivity information) than a nodal affinity model.
